# Supplementary material for: The Chlorophyll Fluorescence Parameter Fv/Fm Correlates with Loss of Grain Yield after Severe Drought in Three Wheat Genotypes Grown at Two CO2 Concentrations
Source: Plants (Basel). 2023 Jan 18;12(3):436. doi: 10.3390/plants12030436 (PMC9920701; doi:10.3390/plants12030436)
Supplement: Supplementary file 1 [file plants-12-00436-s001.zip › plants-2132286-Supplementary Figures.pdf]

Supplementary Materials

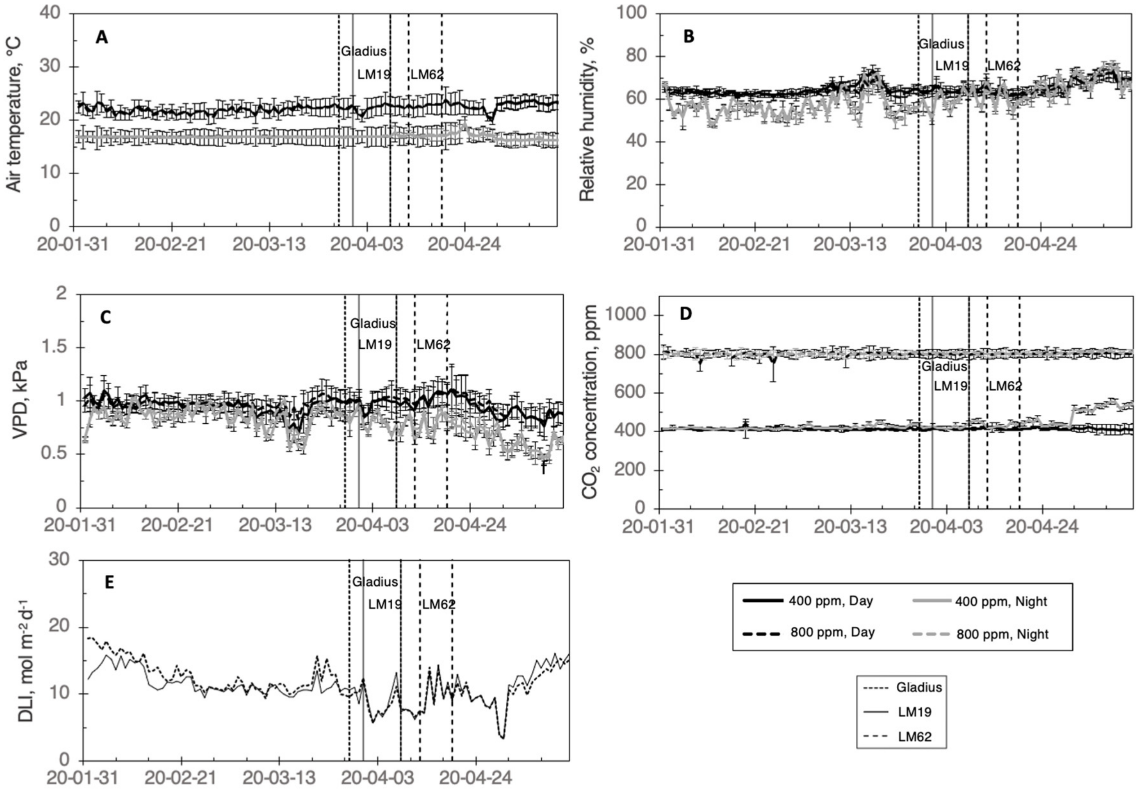

Suppl. Figure S1: Climate conditions within the greenhouse compartments (cells) during the growth period. The figures depict the greenhouse cells' day-time (black lines) and night-time (light grey lines) values. Figure **A** shows the temperature in °C, **B** shows the relative humidity in percent, **C** shows the vapour pressure deficit (VPD) in kPa and **D** shows the CO_2 concentration in ppm. Figure **E** shows the daily light integral in mol m^{-2} d^{-1} where solid lines depict the conditions in the greenhouse cell at 400 ppm CO_2, while dotted lines depict the conditions in the greenhouse cell at 800 ppm CO_2. All data is from the growth period (March 20 – May 11, 2020). The timing of treatment of either genotype is defined as boxes named either Gladius (vertical dots in dotted lines), LM19 (vertical straight lines), or LM62 (dots in vertical lines shown as small lines). The data are mean values ± SD, n = every 3 minutes between 06 and 22 for daytime and between 22 and 06 for night-time.

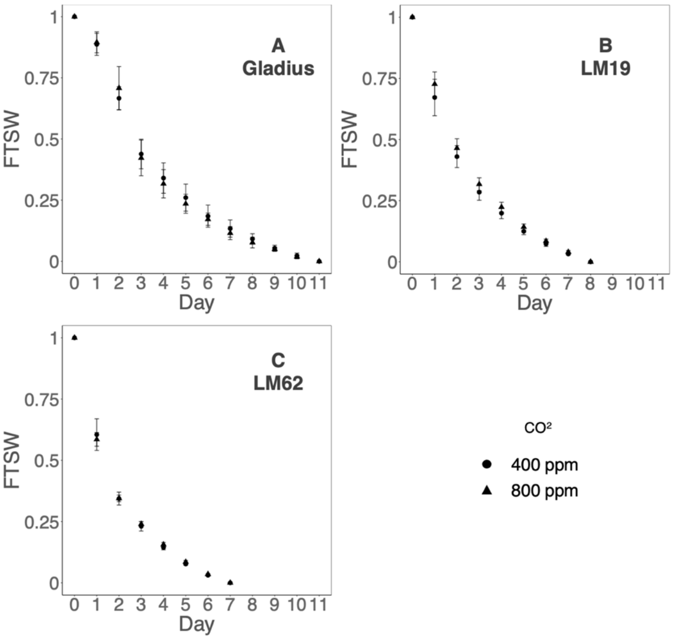

Suppl. Figure S2: Fraction of transpirable water content (FTSW) left in the pot calculated according to eq. 1 for the three genotypes; Gladius (**A**), LM19 (**B**) and LM62 (**C**) throughout the drying cycle (n = 4-7).

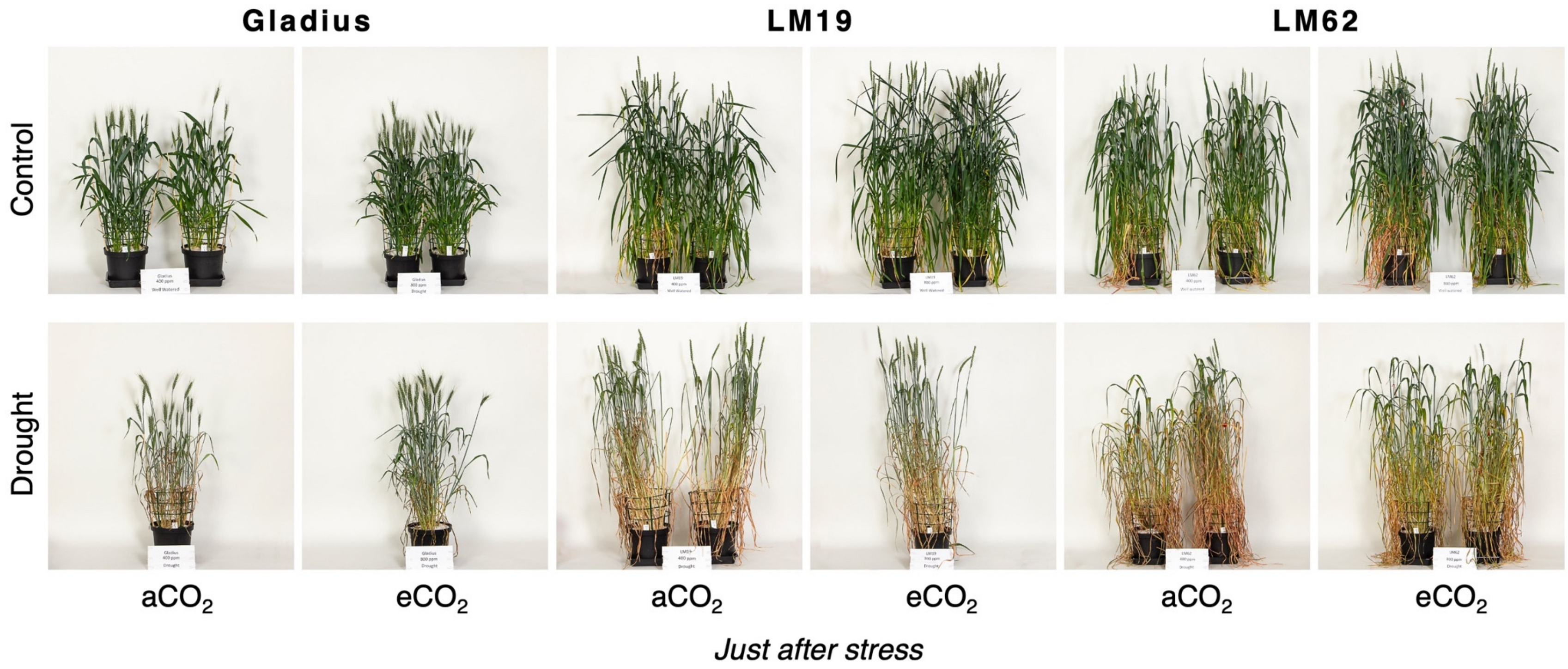

Suppl. Figure S3: The state of the control and drought treatment groups of each genotype after stress. The upper row shows the control group, while the lower row shows the drought-treated plants. Each row is divided into each genotype, from left to right: Gladius, LM19 and LM62. In addition, each genotype is divided into aCO_2 (left)and eCO_2 (right).

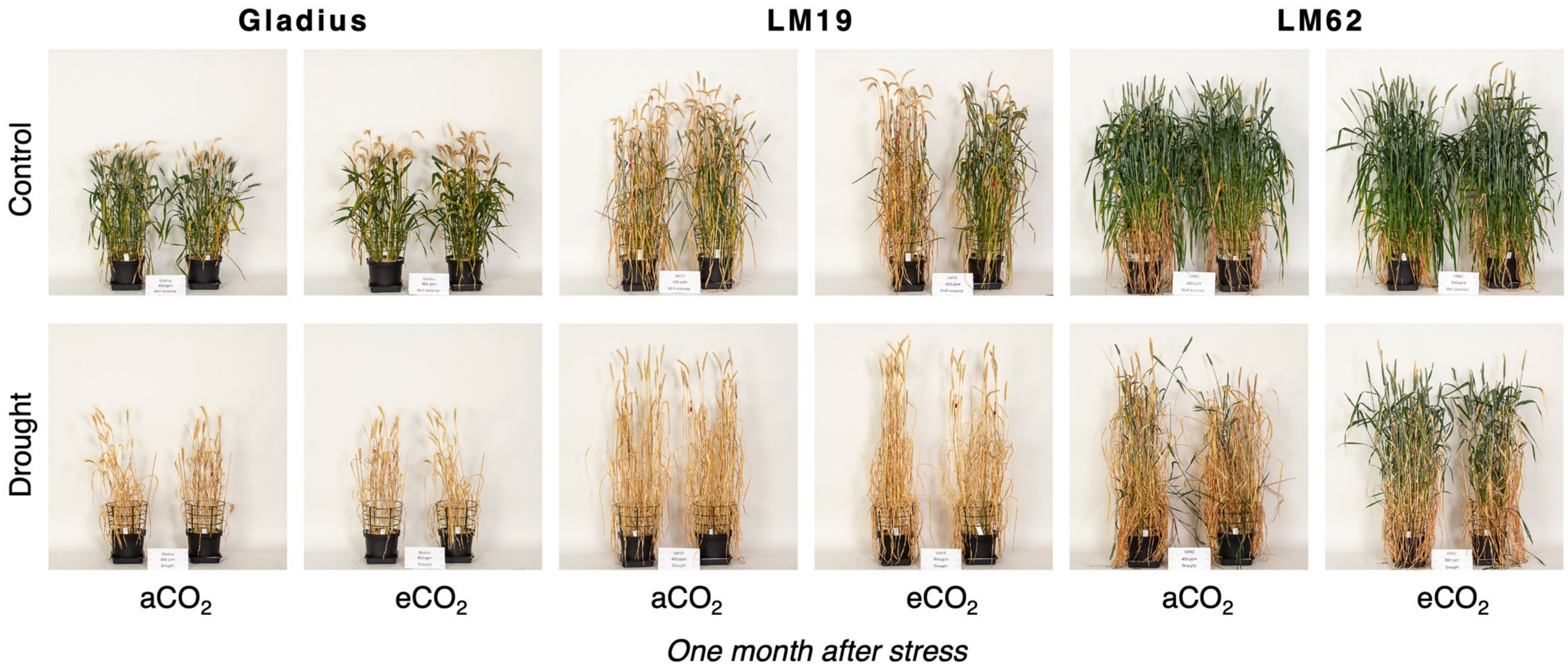

Suppl. Figure S4: The state of the control and drought treatment groups of each genotype one month after the last day of stress for LM62. The upper row shows the control group, while the lower row shows the drought-treated plants. Each row is divided into each genotype, from left to right: Gladius, LM19 and LM62. Each genotype is divided into aCO_2 (left) and eCO_2 (right).

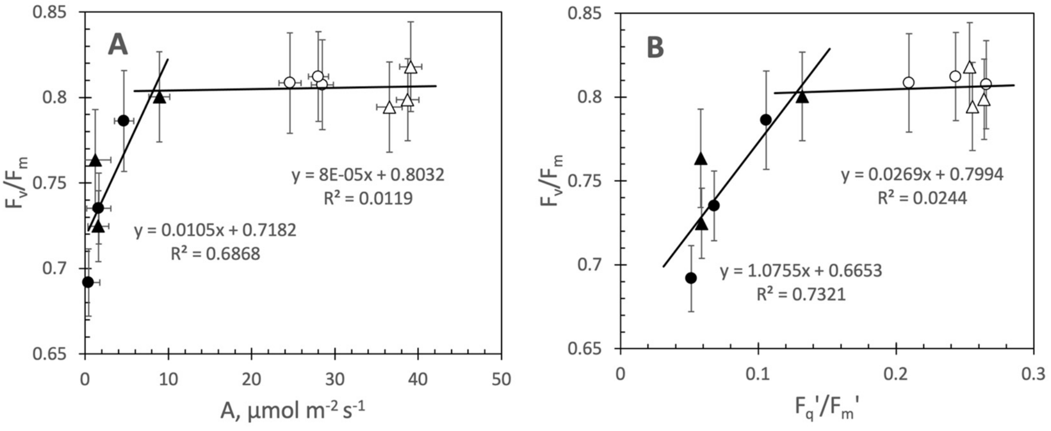

Suppl. Figure S5: The relation between (**A**) F_v/F_m and net photosynthetic rate (A), and (**B**) F_v/F_m and F_q'/F_m' in control and drought-treated plants. White symbols depict control conditions, while black symbols depict drought conditions. Circles are from aCO_2, while triangles are from eCO_2 conditions. The lines represent the linear regression for the control and drought treatments. Values are mean ± SE (n = 4 for A and F_q'/F_m', and n = 8-16 for F_v/F_m).

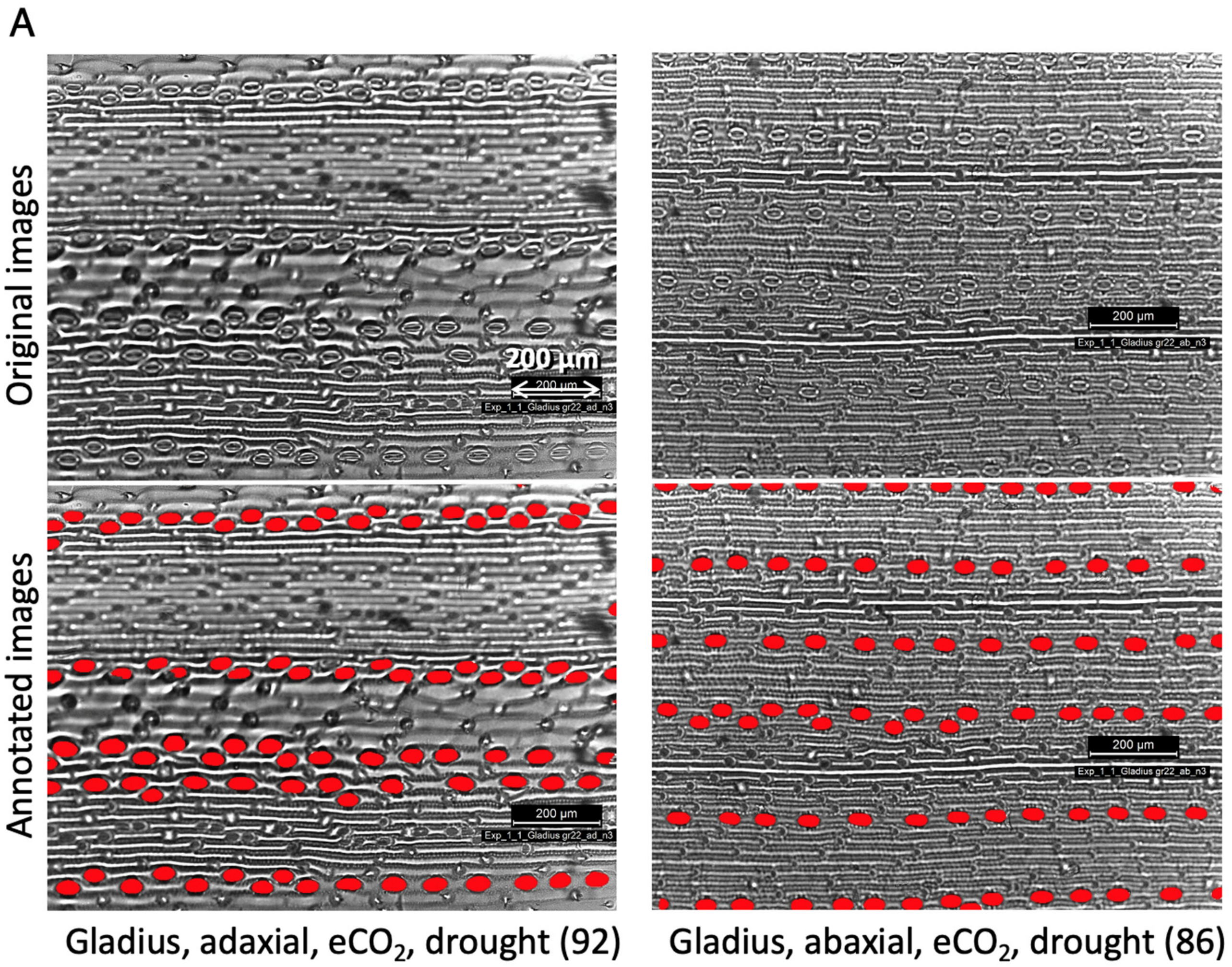
A
Original images
Annotated images
200 µm
200 µm
Exp_1_1_Gladius gr22_ad_n3
Exp_1_1_Gladius gr22_ab_n3
Gladius, adaxial, eCO_2, drought (92)
Gladius, abaxial, eCO_2, drought (86)

B

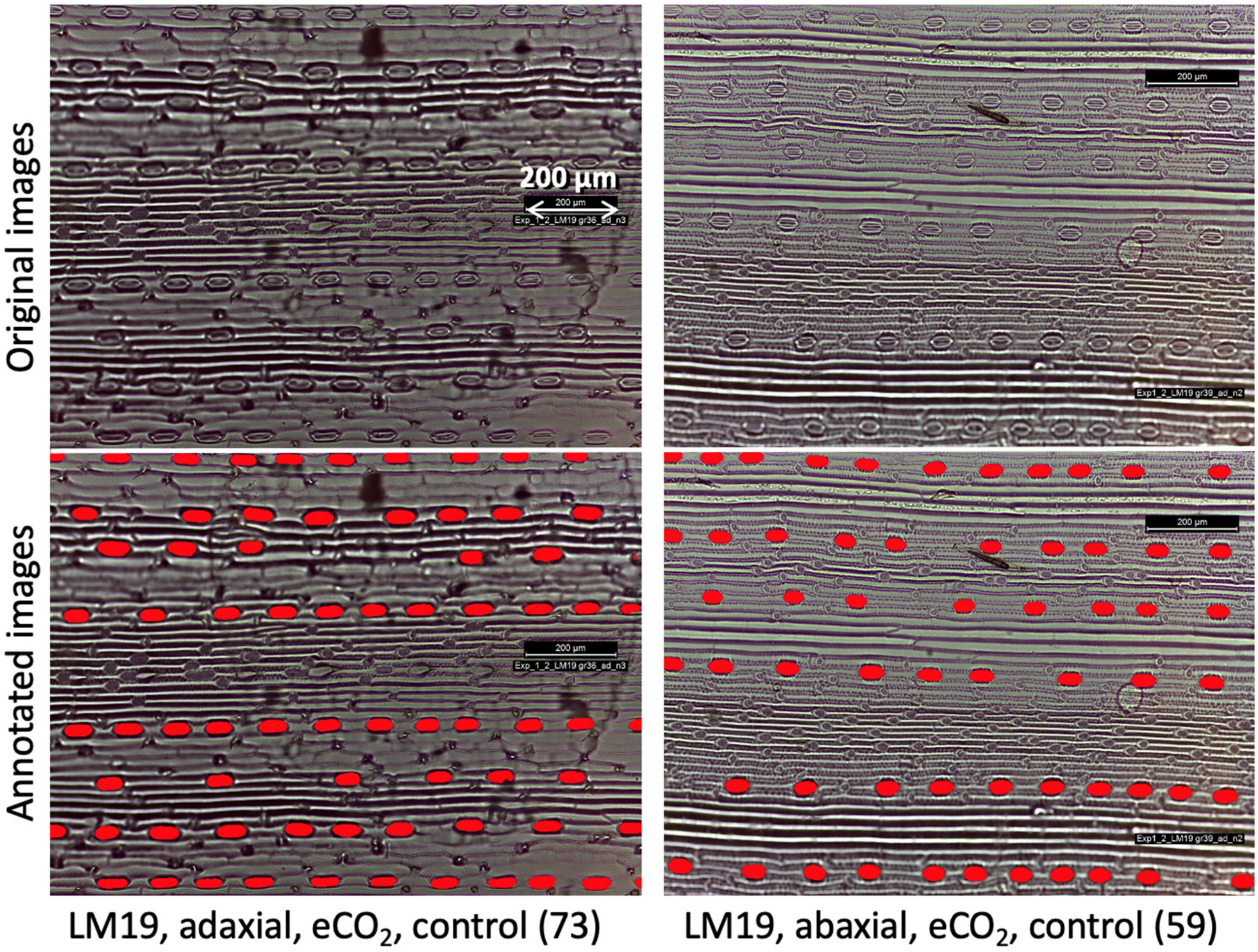

Original images
Annotated images
200 µm
200 µm
Exp_1_2_LM19 gr36_ad_n3
Exp1_2_LM19 gr39_ad_n2
LM19, adaxial, eCO_2, control (73)
LM19, abaxial, eCO_2, control (59)

C

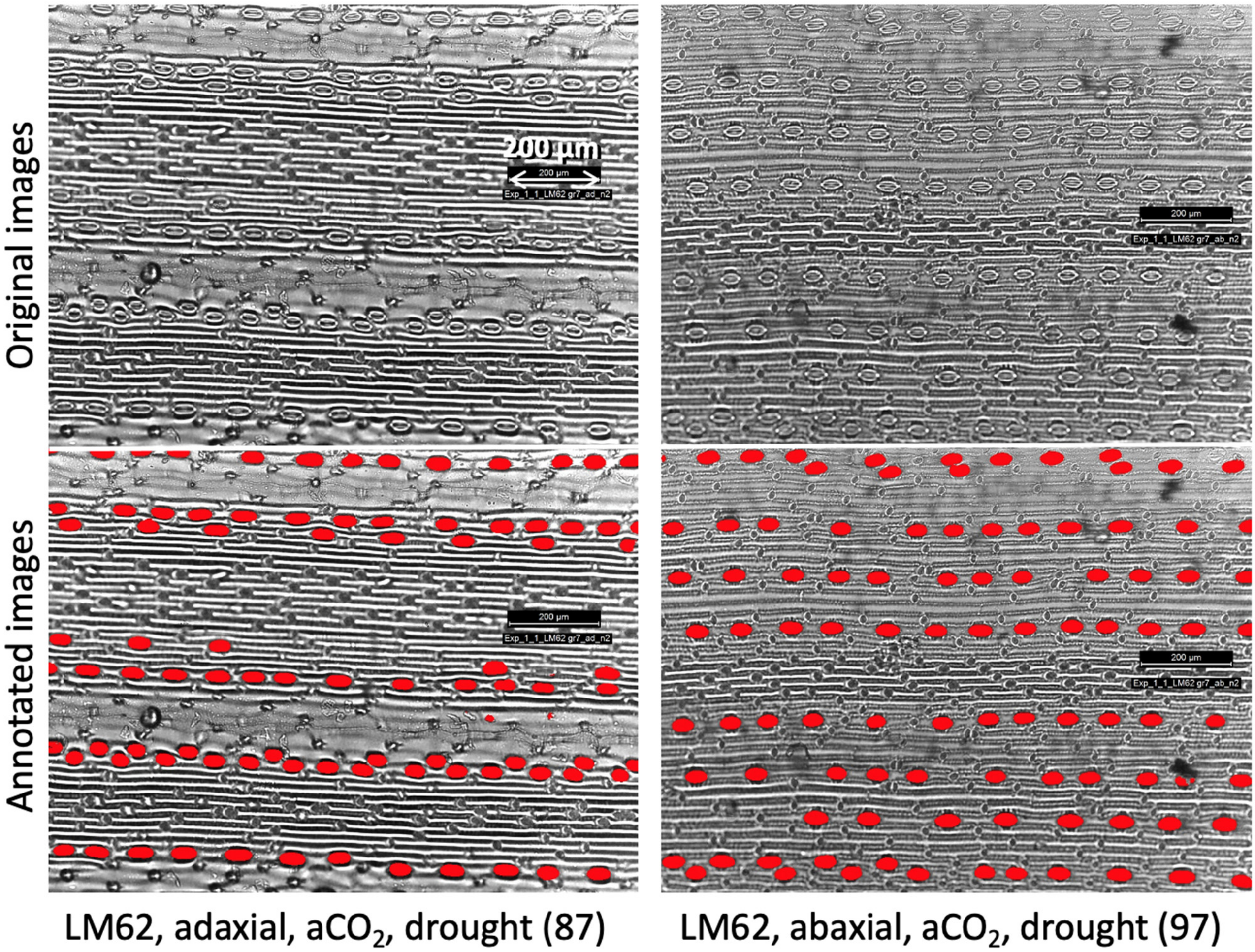

Suppl. Figure S6: Pictures taken with a Leica DM 750 stereomicroscope of Gladius (**A**), LM19 (**B),** and LM62 **(C)**. Pictures on the top rows are original images, and the corresponding images below are shown with segmented stomates (in red) using the trained model. Each column shows a different replicate chosen randomly, with emphasis on different quality and contrast.
